# A Longitudinal Assessment of Two Suicide Prevention Training Programs for the Construction Industry

**DOI:** 10.3390/ijerph17030803

**Published:** 2020-01-28

**Authors:** Victoria Ross, Neil Caton, Jorgen Gullestrup, Kairi Kõlves

**Affiliations:** 1 Australian Institute for Suicide Research and Prevention, School of Psychology, Griffith University, Brisbane 4122, Australia; n.caton@griffith.edu.au (N.C.); k.kolves@griffith.edu.au (K.K.); 2 MATES in Construction, Lvl 1/35 Astor Terrace, Spring Hill QLD 4004, Australia; jorgen@micqld.org.au

**Keywords:** suicide prevention, construction workers, training, evaluation

## Abstract

As part of a suite of early intervention training and support services, Mates in Construction (MATES) provide two general awareness programs to promote mental health and suicide awareness and encourage help-offering and help-seeking in construction workers. General awareness training (GAT) is a one-hour session delivered to all construction workers on large to medium worksites, while MATES awareness training (MAT) maintains similar content but is of shorter duration and delivered informally to small workplaces. This study aimed to compare the effectiveness of the two programs using a before, after and follow-up design. Construction workers undertaking MAT or GAT training completed a short survey before and after their training and again at follow-up. Linear mixed-effect modelling indicated that GAT and MAT training provided similar results in improving suicide awareness and help-seeking intentions. Some variables showed a significant increase from pre-intervention to the three-month follow-up, indicating the long-term impact of some aspects of the training. The findings demonstrating the effectiveness of MAT training have important implications for MATES, as the training can be delivered to much smaller workplaces, making the program more widely available to the construction industry.

## 1. Introduction

Suicide rates differ across industry and occupational groups, with the construction sector identified as one of the highest occupational risk groups for suicide in Australia [1,2,3]. Mates in Construction (MATES) was designed to address these high suicide rates through the implementation of a multi-component prevention and early intervention program. As part of a suite of MATES suicide prevention training and support programs (which include a suicide prevention hotline, field officers and case management support to workers and their families), suicide prevention training is provided in the form of gatekeeper training (GKT). GKT aims to enable non-specialists to identify and respond to those at risk and is one of the most common forms of suicide prevention training [4]. Evidence suggests that GKT is effective in reducing suicide as part of a systematic approach to suicide prevention. It has been adopted in many community suicide-prevention strategies worldwide (e.g., European Alliance Against Depression, Lifespan): however, concerns exist about the long-term effectiveness of training outcomes [5]. Two of MATES’ primary intervention programs were specifically designed to promote awareness about mental health and warning signs for suicidality within the construction industry: general awareness training (GAT) and more recently, MATES awareness training (MAT). Both MAT and GAT are provided at no out-of-pocket cost to employers or workers, although training is always delivered during working hours.

GAT is a one-hour session delivered to all construction workers on large to medium worksites. It is delivered by a minimum of two trainers to groups of between 20 and 300 workers using a PowerPoint presentation. GAT is designed to reduce the stigma associated with suicide, encourage help-offering and help-seeking, and present suicide as a preventable workplace health and safety issue for the construction industry. Workers are taught how to identify the warning signs for suicidality and are encouraged to offer active support to struggling co-workers. Previous pre–post training evaluation research has shown GAT to be an effective program in fostering suicide prevention awareness [6] and shifting attitudes towards suicide and mental health [7]. In addition, the overall MATES program has been found to increase help-seeking behaviours and reduce stigma around mental health issues in the mining industry [8].

MAT maintains similar content to GAT but is of only 15 min duration and delivered by a single trainer using an informal, conversational structure. It was designed to be delivered in small workplaces with up to 20 workers, where it would be impracticable to deliver the full GAT used on larger sites. MAT is delivered without any audio/visual technology (which is not practical on small sites) and has a conversational focus on how workers can look out for each other. To date, however, there has been no empirical evidence to support the effectiveness of MAT. Given the shorter duration of MAT (and thus potential time and cost savings), it is important to compare the effectiveness of these programs in improving suicide awareness and help-seeking. Therefore, the aim of this study was to compare MAT and GAT on improving suicide awareness and knowledge, help-offering and help-seeking. Moreover, as there has been no longitudinal evidence for the effectiveness of these programs in the construction industry, the present study will investigate the effectiveness of MAT and GAT using a longitudinal study design.

## 2. Materials and Methods

### 2.1. Study Design and Data Collection

A longitudinal before, after and follow-up design was applied to examine the effectiveness of MAT and GAT training. Construction workers undertaking MAT or GAT training between March and June 2018 at construction sites across Queensland, Australia, were asked to complete a short paper-based survey before (i.e., on arrival at the training session) and after their training (intervention), and again at follow-up. For GAT, 2260 participants (*M_age_* = 34.94 ±.77; 91.8% males) completed the pre-, 2241 post- (*M_age_* = 34.89 ± 15.80; 91.8% males) and 189 (8.4%) follow-up surveys (*M_age_* = 41.73 ± 14.57; 83.4% male); and for MAT, 717 (*M_age_* = 30.56 ± 19.82; 95.5% males), 700 (97.6%) (*M_age_* = 30.43 ± 19.62; 95.9% males), and 56 (7.8%) (*M_age_* = 37.43 ± 19.17; 91.1% males) participants completed the pre-, post-, and follow-up surveys, respectively. Follow-up surveys were completed on-line using a link distributed by SMS between three and six months after the intervention (between September and November 2018).

### 2.2. Outcome Measures

The surveys consisted of six items measuring suicide awareness and knowledge, attitudes to help-seeking and giving, which were summed to form an overall *suicide awareness* measure (pre-intervention: *α* = 0.77; post-intervention: *α* = 0.78; follow-up: *α* = 0.73); and one question measuring emotional well-being (1 = very poor, 2 = poor, 3 = OK, 4 = good, 5 = very good). The help-seeking item: *If I was going through a difficult time*, *feeling upset, or was thinking about suicide*, *I would be willing to seek help* and list of response options (e.g., *intimate partner*, *friend, doctor*) was adapted from the General Help-Seeking Questionnaire [9]. These 12 items were also summed to create an overall *help-seeking intentions* measure (pre-intervention: *α* = 0.84; post-intervention: *α* = 0.84; follow-up: *α* = 0.75). All items required responses on a five-point Likert scale from 1 = strongly disagree to 5 = strongly agree. The study was approved by the Griffith University’s Human Research Ethics Committee (GU Reference number 2017/353).

### 2.3. Statistical Analysis

Linear mixed-effect modelling was used to analyse the effectiveness of the intervention for suicide awareness, help-seeking intentions, and emotional well-being. Linear mixed-effect modelling is a statistical technique for examining longitudinal data that accounts for both within- and between-subjects variance, as well as the correlation between the repeated measures of each participant [10]. Importantly, this technique manages unbalanced data with the assumption that missing data are missing at random and are not excluded from the analyses. Considering the high attrition rate from post-intervention to follow-up for both MAT (i.e., 92.0% attrition) and GAT (i.e., 91.6% attrition), sensitivity analyses were conducted using multiple imputation, which did not alter our results. This corroborates Peters et al. [11] who demonstrated that multiple imputation does not change the results for repeated-measures linear mixed models. Thus, the imputed dataset was not included in the final analysis.

All variables, except, *If my mate was going through a difficult time feeling upset or thinking about suicide*, *I would be willing to offer help*, were normally distributed (the range for skewness or kurtosis below +1.5 and above −1.5 [12]). The appropriate transformations were applied to the non-normally distributed variable, as it did not change the results, the untransformed variable was used. Nonetheless, linear mixed-effect models are arguably robust against violations of normality [13].

For the linear mixed-effect models, intervention type (GAT and MAT), time (pre, post, and follow-up), date on which the intervention was undertaken, training session (the group that undertook the intervention together, sometimes on the same date), age and gender were entered as fixed effects. By controlling for the intervention date, the difference in follow-up time points is ultimately controlled (i.e., as follow-up data was collected between three and six months after the intervention). The participant ID variable was included in the random intercept to model for within-person factors at baseline. First-Order Autoregressive (AR1) and Unstructured (UN) covariance structures were examined using −2 Res Log Likelihood and Akaike’s Information Criterion (AIC). Both structures were applied to the levels of training session*participant ID (as both MAT and GAT were delivered in training sessions at different locations, therefore participants were nested within groups). The UN structure was identified as the model with the best fit with all dependent variables. Post-hoc analyses for the linear mixed models were conducted with Sidak adjustment. An alpha level of 0.05 was employed. All analyses were performed with the IBM SPSS version 25.0 (IBM Corp., Armonk, NY, USA).

## 3. Results

### 3.1. Suicide Awareness

A significant time effect emerged for the overall measure and five of the six suicide awareness items. However, there were no significant effects of intervention type (GAT vs MAT) nor the interaction (Table 1). Post-hoc analyses were then analysed for measures that exhibited a significant time effect (Table 2). The overall measure and the five suicide awareness items showed a significant increase from pre- to post-intervention, but only the item, *If my workmate was going through a difficult time, feeling upset or thinking about suicide*, *I would know how to connect him/her to appropriate help* significantly increased from pre-intervention to follow-up.

**Figure 1:**
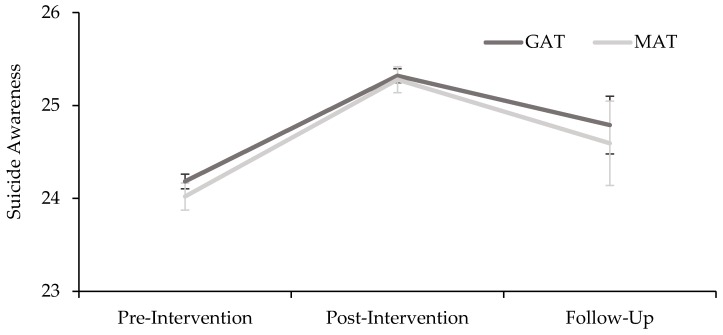
Estimated marginal means for intervention (general awareness training (GAT) and MATES awareness training (MAT)) and time (pre, post, and follow-up) on suicide awareness. Error bars represent standard errors.

Post-hoc results for overall suicide awareness are depicted in Figure 1.

### 3.2. Help-Seeking Intentions

A significant time effect occurred for the overall help-seeking intention measure, and 10 of the 12 help-seeking intention items. There were no significant effects for intervention type (GAT vs MAT), and there was one significant effect for the interaction (i.e., Minister/religious leader; Table 3).

Post-hoc analyses for time and help-seeking intentions are presented in Table 4. Three response items, *MATES worker/connector*, *Telephone helpline* and *Seek help from another*, significantly increased from pre-intervention to follow-up. The overall measure and the nine help-seeking intention items showed a significant increase from pre- to post-intervention, but this was not maintained to follow-up. One item (i.e., Close family) significantly decreased from post-intervention to follow-up.

**Figure 2:**
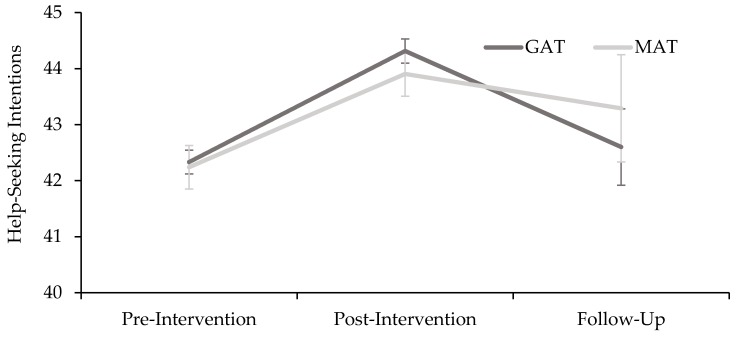
Estimated marginal means for intervention (GAT and MAT) and time (pre-, post-, and follow-up) on help-seeking intentions. Error bars represent standard errors.

Post-hoc results for overall help-seeking intentions are presented in Figure 2.

### 3.3. Well-Being

Emotional well-being was significantly predicted by time (F_2,992.91_ = 14.15, *p* < 0.001), but not by intervention type (F_1,438.90_ = <0.001, *p* = 0.99), nor the interaction (F_2,464.42_ = 0.78, *p* = 0.46). Post-hoc analyses indicated a significant improvement in well-being from pre- to post-intervention (*p* < 0.001, mean difference = −0.05, 95%CI:−0.07,−0.03), but not from pre-intervention to follow-up (*p* = 0.67, mean difference = 0.10, 95%CI:−0.13,.33) nor from post-intervention to follow-up (*p* = 0.35, mean difference = 0.15, 95%CI:−0.09,.38).

## 4. Discussion

This study is the first to longitudinally investigate the effectiveness of MATES suicide prevention training in the construction industry from pre, post to three months follow-up. Notably, the results from our study showed no intervention effect, indicating that MAT which is of shorter duration and delivered in a more informal style, was not significantly different from GAT with regards to the outcome measures. For both MAT and GAT there was a significant time effect for the overall suicide awareness measure and five of its items, including help-offering and help-seeking items. Post hoc analysis of time effect showed significant improvements from pre- to post-intervention and the item, *If my workmate was going through a difficult time*, *feeling upset, or thinking about suicide*, *I would know how to connect him/her to appropriate help*, maintained this significant increase to the three month follow-up. This suggests a long-term impact for the aspect of training on how to connect workmates to appropriate help.

A similar pattern was found for the response options for intentions to seek help. All help-seeking responses (except *Doctor*) showed a significant increase from pre- to post-intervention. In addition, the three response items of *MATES worker/connector, Telephone helpline*, and *seek help from another* all maintained a significant increase from pre-intervention to follow-up, indicating the long-term impact of this aspect of the training. It is plausible that the significant decrease in the response option, *Close family*, could be due to the other options presented in the training (e.g., individuals who considered close family as their only option for asking for help, become aware of other options after training).

Overall, these findings showing significant improvements from pre- to post-training, are consistent with previous evaluation research which found support for the effectiveness of MATES training [7,14]. Nevertheless, those studies were limited to GAT training and did not include follow-up. Sayers et al. [8], found some evidence for long-term change in help-seeking behaviours in the mining industry after MATES in mining training (including GAT); however, they were not able to match participants across the three data collection time points and thus unable to complete a longitudinal analysis.

These results have important implications for MATES suicide prevention training, as this will allow the delivery of MATES training to much smaller workplaces in the industry, thus making the program available more widely to the construction industry. MATES would need to examine the implications of making the program more widely available and the requirement of further support services for those identified to be at risk of suicide. Workers engaged in smaller workplaces are generally more isolated and have access to less services than those on larger sites, thus more training on smaller sites could result in the need for increased resources such as case management support. Although our results showed support for some aspects of training over time, the findings suggest that the impact of GAT and MAT does generally lessen over time, indicating the need for refresher training.

A limitation of this study was the high rate of attrition at follow-up, although this was addressed using linear mixed-effects regression. Sensitivity analyses also indicated that multiple imputation did not change our results. Future research would benefit from strategies to improve participant retention [15]. Other limitations included the lack of a control group (which would have enabled analysis for potential biases) and the use of a convenience sample; however, it should be noted that it is difficult to randomise participants in this type of *real-world* setting.

## 5. Conclusions

This longitudinal study indicated that GAT and MAT training were equally effective in improving suicide awareness and knowledge, help-offering and help-seeking after training. The results also showed a time effect, with some variables showing a significant increase from pre-intervention at the three- to six-month follow-up, indicating the long-term impact of some aspects of the training. It will be important to continue to observe whether these impacts lessen over time. Further research may also be useful to assist in understanding the impact of additional MATES support services and materials (e.g., MATES Connectors, toolbox talks) on the long-term effectiveness of MAT and GAT training.

## Figures and Tables

**Table 1:** Fixed effect estimates for suicide awareness.

	Time			Intervention Type			Time*Intervention		
	*F*	*df ^1^*	*p*	*F*	*df ^1^*	*p*	*F*	*df ^1^*	*p*
Suicide awareness score.	152.24	876.2	<0.001	0.88	679.7	0.35	0.40	482.4	0.67
Talking about suicide can prevent suicide.	28.88	1003.1	<0.001	<0.01	498.5	0.98	0.46	489.7	0.63
If my workmate was going through a difficult time feeling update or thinking about suicide, I think I would notice.	77.89	999.0	<0.001	0.40	497.2	0.53	2.20	485.7	0.11
If my mate was going through a difficult time feeling upset or thinking about suicide, I would be willing to offer help.	0.68	979.5	0.51	2.40	547.7	0.12	2.01	506.9	0.13
If my workmate was going through a difficult time feeling up set or thinking about suicide, I would know how to connect him/her to appropriate help.	226.44	1016.0	<0.001	1.93	475.4	0.17	0.08	488.9	0.92
My current worksite supports good mental health and well-being.	47.54	994.1	<0.001	0.86	505.3	0.35	1.39	481.8	0.25
If I was going through a difficult time, feeling upset, or was thinking about suicide, I would be willing to seek help.	60.43	955.7	<0.001	<0.01	551.2	0.98	1.56	484.4	0.21

Note. ^1^ = denominator degrees of freedom. Numerator degrees of freedom are as follows: Time (2), Intervention (1), Time*Intervention (2).

**Table 2:** Linear mixed-effect model post-hoc analyses on time for suicide awareness.

	Pre- to Post-Intervention			Post- to Follow-up			Pre- to Follow-Up		
	Mdif	*p*	95% CI	Mdif	*p*	95% CI	Mdif	*p*	95% CI
Suicide awareness score.	−1.20	<0.001	[−1.36,−1.03]	0.61	0.22	[−0.23,1.44]	−0.59	0.25	[−1.43,0.25]
Talking about suicide can prevent suicide.	−0.14	<0.001	[−0.18,−0.09]	0.02	0.99	[−0.19,0.24]	−0.12	0.48	[−0.33,0.10]
If my workmate was going through a difficult time feeling update or thinking about suicide, I think I would notice.	−0.24	<0.001	[−0.28,−0.19]	0.19	0.09	[−0.02,0.41]	−0.05	0.95	[−0.26,0.17]
If my mate was going through a difficult time feeling upset or thinking about suicide, I would be willing to offer help ^1^.	-	-	-	-	-	-	-	-	-
If my workmate was going through a difficult time feeling up set or thinking about suicide, I would know how to connect him/her to appropriate help.	−0.43	<0.001	[−0.48,−0.38]	0.05	0.91	[−0.16,0.26]	−0.38	<0.001	[−0.59,−0.17]
My current worksite supports good mental health and well-being.	−0.16	<0.001	[−0.20,−0.12]	−0.01	0.99	[−0.26,0.24]	−0.18	0.26	[−0.43,0.08]
If I was going through a difficult time, feeling upset, or was thinking about suicide, I would be willing to seek help.	−0.20	<0.001	[−0.24,−0.16]	0.13	0.43	[−0.10,0.36]	−0.07	0.87	[−0.30,0.17]

Note. ^1^ = post-hoc analyses on time were not run as this item did not exhibit a main effect for time; Mdif—mean difference. A negative and positive mean difference indicates an increase and decrease, respectively.

**Table 3:** Fixed effect estimates for help-seeking intention.

Help-Seeking Source	Time			Intervention Type			Time*Intervention		
	*F*	*df ^1^*	*p*	*F*	*df ^1^*	*p*	*F*	*df ^1^*	*p*
Help-seeking intentions	75.00	674.0	<0.001	0.02	990.5	0.88	1.21	497.9	0.30
Intimate partner	25.26	911.1	<0.001	1.29	593.6	0.26	0.37	474.3	0.68
Close family	32.38	886.3	<0.001	0.08	628.9	0.78	0.19	464.3	0.83
Friend	41.86	988.5	<0.001	1.59	485.7	0.21	0.15	489.0	0.86
Workmate	99.23	899.05	<0.001	1.21	581.7	0.27	2.34	476.3	0.09
Supervisor	46.95	891.3	<0.001	0.62	572.0	0.43	1.02	466.4	0.36
Doctor	2.64	855.3	0.07	1.27	618.3	0.26	1.01	457.6	0.37
MH professional	18.83	842.2	<0.001	1.37	637.9	0.24	1.07	455.3	0.35
Telephone helpline	77.59	893.4	<0.001	3.03	597.4	0.08	0.48	472.7	0.62
MATES worker/connector	155.98	905.9	<0.001	0.06	566.2	0.82	0.74	482.9	0.48
Minister/religious leader	17.21	733.2	<0.001	0.29	885.86	0.59	3.84	447.4	0.02
Seek help from another	12.71	864.5	<0.001	0.13	583.40	0.72	1.19	511.0	0.31

Note. ^1^ = denominator degrees of freedom. Numerator degrees of freedom are as follows: Time (2), Intervention (1), Time*Intervention (2).

**Table 4:** Linear mixed model post-hoc analyses on time for help-seeking intention.

Help-Seeking Source	Pre- to Post-Intervention			Post- to Follow-Up			Pre- to Follow-Up		
	*Mdif*	*p*	*95% CI*	*Mdif*	*p*	*95% CI*	*Mdif*	*p*	*95% CI*
Help-seeking intentions	−1.82	<0.001	[−2.18, −1.47]	1.16	0.37	[−0.74, 3.07]	−0.66	0.79	[−2.57, 1.25]
Intimate partner	−0.10	<0.001	[−0.13, −0.06]	0.23	0.08	[−0.02, 0.49]	0.14	0.48	[−0.12, 0.40]
Close family	−0.11	<0.001	[−0.15, −0.08]	0.31	0.01	[0.06, 0.57]	0.20	0.17	[−0.06, 0.45]
Friend	−0.15	<0.001	[−0.19, −0.11]	0.24	0.08	[−0.02, 0.50]	0.09	0.81	[−0.17, 0.34]
Workmate	−0.28	<0.001	[−0.33, −0.23]	0.18	0.32	[−0.10, 0.46]	−0.10	0.78	[−0.38, 0.18]
Supervisor	−0.20	<0.001	[−0.24, −0.15]	0.08	0.91	[−0.24, 0.39]	−0.12	0.75	[−0.43, 0.20]
Doctor ^1^	-	-	-	-	-	-	-	-	-
MH professional	−0.12	<0.001	[−0.17, −0.07]	−0.16	0.49	[−0.45, 0.14]	−0.28	0.07	[−0.57, 0.02]
Telephone helpline	−0.28	<0.001	[−0.34, −0.23]	−0.08	0.89	[−0.39, 0.23]	−0.36	0.01	[−0.67, −0.06]
MATES worker/connector	−0.40	<0.001	[−0.45, −0.34]	−0.19	0.25	[−0.46, 0.08]	−0.59	<0.001	[−0.86, −0.32]
Minister/religious leader ^2^	-	-	-	-	-	-	-	-	-
Seek help from another	−0.13	<.0001	[−0.20, −0.06]	−0.23	0.30	[−0.58, 0.12]	−0.36	0.04	[−0.71, −0.02]

Note. ^1^ = post-hoc analyses on time were not run as this item did not exhibit a main effect for time. ^2^ = as there was a significant interaction, post-hoc analyses were conducted on the interaction. Mdif—mean difference. A negative and positive mean difference indicates an increase and decrease, respectively.
